# In the business of base editors: Evolution from bench to bedside

**DOI:** 10.1371/journal.pbio.3002071

**Published:** 2023-04-12

**Authors:** Elizabeth M. Porto, Alexis C. Komor

**Affiliations:** Department of Chemistry and Biochemistry, University of California, San Diego, La Jolla, California, United States of America

## Abstract

With the advent of recombinant DNA technology in the 1970s, the idea of using gene therapies to treat human genetic diseases captured the interest and imagination of scientists around the world. Years later, enabled largely by the development of CRISPR-based genome editing tools, the field has exploded, with academic labs, startup biotechnology companies, and large pharmaceutical corporations working in concert to develop life-changing therapeutics. In this Essay, we highlight base editing technologies and their development from bench to bedside. Base editing, first reported in 2016, is capable of installing C•G to T•A and A•T to G•C point mutations, while largely circumventing some of the pitfalls of traditional CRISPR/Cas9 gene editing. Despite their youth, these technologies have been widely used by both academic labs and therapeutics-based companies. Here, we provide an overview of the mechanics of base editing and its use in clinical trials.

## Abstract 2

Base editing, first reported in 2016, is a genome editing technology capable of installing point mutations with high efficiency and precision. This Essay discusses base editing technologies and their development from bench to bedside.

## Introduction

Precision medicine has long been a major focus of biological application-based research, and the development of CRISPR-derived genome editing tools has propelled progress in this area forward in recent years. In particular, base editors have demonstrated their worth as especially powerful tools for the development of genome editing therapies. Base editing technologies were derived from CRISPR/Cas9 systems but avoid the use of double-strand breaks (DSBs) that traditional genome editing systems use. Bypassing the use of DSBs largely prevents the introduction of stochastic genome editing byproducts (such as indels). However, the trade-off for this enhancement in genome editing precision is that base editors can only perform certain types of single base pair edits (transition mutations—purine to purine or pyrimidine to pyrimidine mutations), rather than the insertion, deletion, or replacement of any stretch of DNA desired. Fortunately, the ability to install transition point mutations with high precision and efficiency can be leveraged for a variety of therapeutical applications (not only the correction of monogenic disease-causing point mutations), making base editors fitting tools for the clinic.

In this Essay, we describe the initial development of base editors and discuss their limitations and the subsequent improvements made to the original base editor constructs. We focus on modifications made to improve the efficiency, precision, and specificity of base editors, particularly in the context of therapeutics. We then provide an overview of the four current base editing clinical trials, focusing on the general genome editing strategies employed by each trial. We finish with a brief commentary on future base editing clinical trials in the immediate pipeline, additional emerging next-generation genome editing tools, and ethical considerations to consider as genome editing therapeutics become more prevalent.

## Base editing technologies

### Cytosine base editors

**Fig 1:**
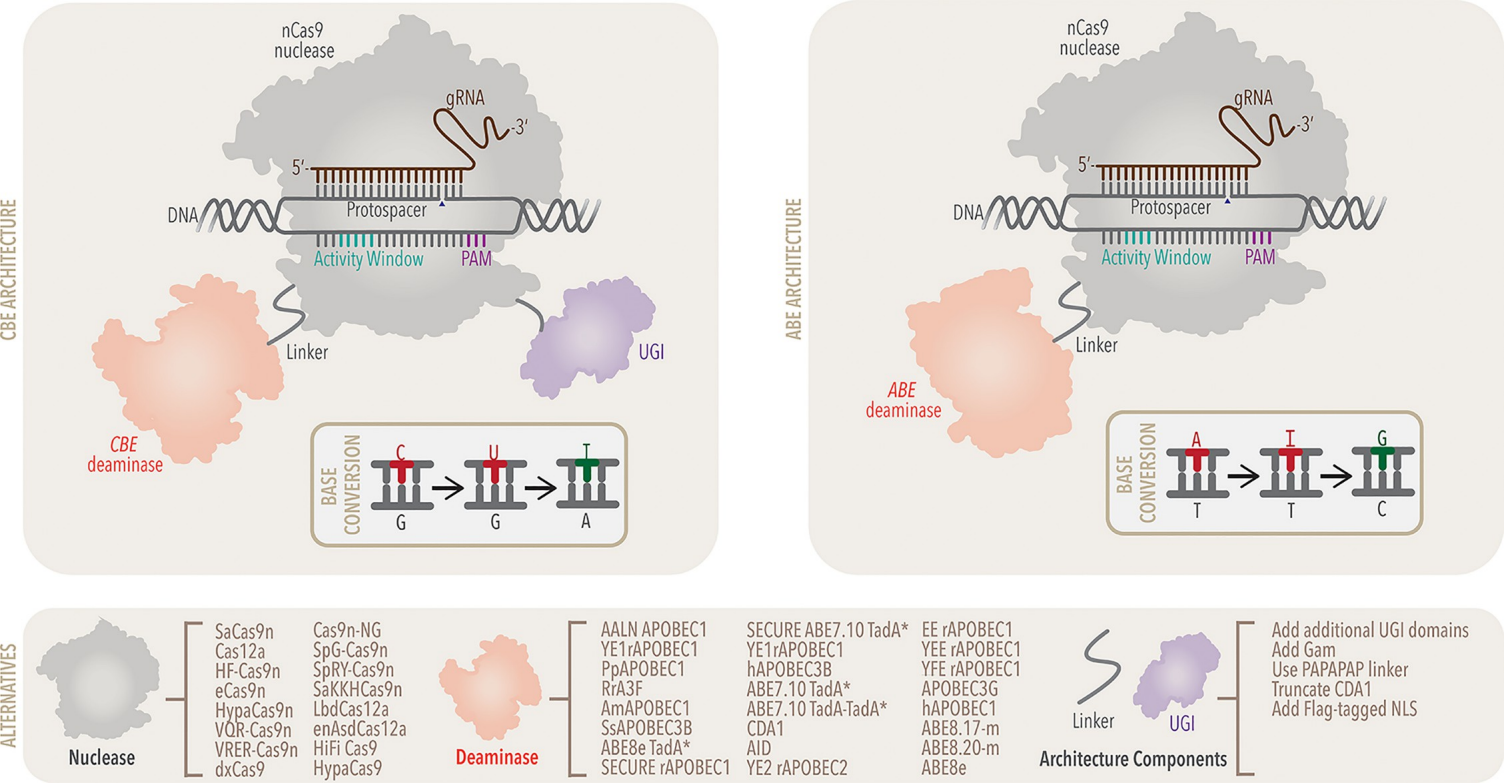
Overview of CBE and ABE principal components. **Top left**: CBE architecture shown with principal components: Cas9n in grey (outline of crystal structure obtained from PDB: 6VPC), CBE deaminase APOBEC3A in red (outline of crystal structure obtained from PDB: 5SWW), and UGI in purple (outline of crystal structure obtained from PDB: 1UGI). The deaminase and UGI components are tethered to nCas9 via short amino acid linkers (grey). Overlayed on top of the principal components is a general schematic of the mechanism of action; the gRNA (brown) will bind to the DNA protospacer (sequence of 20 nucleotides proximally located to the 3-nucleotide PAM (violet) sequence), in the process exposing a single-stranded DNA “bubble” open for cytosine deamination. Deamination produces a U•G intermediate, which is processed by the cell to produce an overall C•G to T•A conversion (shown in base conversion inset). Concurrently, nCas9 will nick the unedited DNA strand (blue triangle) to increase editing efficiency. Similarly, the addition of the UGI component increases editing efficiency. **Top right**: ABE architecture, simplified mechanism schematic, and overall base conversion are shown. Key differences of the ABE architecture are as follows: ABE deaminase TadA-8e, similarly in red, (outline of crystal structure obtained from PDB: 6VPC) replaces CBE deaminase and the lack of a UGI component, as ABE utilizes an inosine intermediate, compared to the CBE architecture. **Bottom**: A noncomprehensive sampling of notable variations on key CBE and ABE principal components are shown. Collectively, these substitute components serve an array of purposes including increased on-target editing, decreased off-target editing, and relaxed PAM requirements for broadened utility. Development of new and enhanced base editor principal components is a populated field of study with new results being published rapidly. ABE, adenine base editor; AID, activation-induced cytidine deaminase; AmAPOBEC1, *Alligator mississippiensis* APOBEC1; APOBEC, Apolipoprotein B mRNA editing enzyme, catalytic polypeptide; CDA1, cytidine deaminase 1 sourced from sea lamprey; Cas9n, Cas9 nickase; CBE, cytosine base editor; dxCas9, catalytically dead expanded PAM Cas9; eCas9, enhanced specificity Cas9; enAsdCas12a, enhanced *Acidaminococcus* sp. BV3L6 catalytically dead Cas12a gRNA, guide RNA; hAPOBEC, human APOBEC; HiFi Cas9, high fidelity Cas9 variant; HF-Cas9, high fidelity Cas9 variant; HypaCas9, hyper accurate Cas9; LbdCas12a, catalytically dead *Lachnospiraceae bacterium* Cas12a; NLS, nuclear localization signal PAM, protospacer adjacent motif; PpAPOBEC1, *Pongo pygmaeus* APOBEC1; rAPOBEC1, *Rattus norvegicus* APOBEC1; RrA3F, *Rhinopithecus roxellana* APOBEC3F; SaCas9, *Staphylococcus aures* Cas9; SECURE, selective curbing of unwanted RNA editing; SpCas9, *Streptococcus pyogenes* Cas9; ssAPOBEC3B, *Sus scrofa* APOBEC3; UGI, uracil glycosylase inhibitor.

Currently, two classes of base editors exist: cytosine base editors (CBEs) and adenine base editors (ABEs). In the first example of targeted point mutation introduction via a non-DSB mechanism, the original CBE (named BE1) was created by fusing a catalytically inactive or “dead” Cas9 (dCas9) enzyme with the naturally occurring cytidine deaminase enzyme APOBEC1 (rAPOBEC1 sourced from *Rattus norvegicus*) [1]. The dCas9 protein complexes with a preprogrammed guide RNA (gRNA) and subsequently locates and binds to a specific DNA sequence (the protospacer) through the formation of an R-loop, driven by base-pairing between the protospacer and the first 20 nucleotides of the gRNA (the spacer; Fig 1) [2]. For the gRNA to bind to the protospacer, the protospacer must also be immediately adjacent to a protospacer adjacent motif (PAM) sequence (Fig 1). In the case of the widely used *Streptococcus pyogenes* Cas9 (spCas9), the PAM sequence is 5′-NGG-3′, which has been calculated to occur once every approximately 42 bases throughout the human genome [3].

Following formation of the Cas9:gRNA:DNA ternary complex, a subset of one DNA strand is now single-stranded and accessible to rAPOBEC1 for deamination chemistry (Fig 1). Cytidines that are within this “editing window” are deaminated by rAPOBEC1, which produces a C•G to U•G conversion. The development and characterization of many subsequent CBEs have revealed that several factors influence which nucleotides within the protospacer comprise this “editing window”, and include the Cas homolog that is used, the linker length and composition between the deaminase and Cas protein, the overall architecture of the base editor, and the deaminase enzyme used (discussed later). For BE1, the deamination activity window is between positions 4 to 8 within the protospacer (Fig 1). Processing of the U•G intermediate by the cell, using the U-containing strand as a template, results in an overall C•G to T•A conversion. However, the presence of the U•G mismatch intermediate triggers the cell’s native base excision repair (BER) pathway to excise the uracil and revert the intermediate back to the original C•G base pair [4]. Consequently, editing activity by BE1 in live mammalian cells was quite low, and C•G to non-T•A conversions were observed as well (discussed later).

To address this, a second-generation CBE was developed, BE2, which incorporated a uracil glycosylase inhibitor (UGI) peptide to temporarily block BER, thus preventing uracil excision and increasing C•G to T•A conversion efficiencies. One last modification to the system was to exchange dCas9 for a nickase version of the enzyme (nCas9) and produced the final original CBE, named BE3. BE3 installs a DNA nick on the strand opposite the uracil-containing strand. This in turn manipulates the cell’s native DNA repair processes to preferentially replace this strand and use the uracil-containing strand as a template, thus increasing editing efficiency even more (Fig 1). Shortly after the development of BE3, an additional CBE (named Target-AID) was described, which included similar components (a cytidine deaminase, nCas9, and UGI), but utilized the more active cytidine deaminase pmCDA1 (cytidine deaminase 1 sourced from sea lamprey) and fused together in a different orientation, resulting in a slightly shifted editing window compared to BE3 [5]. Target-AID demonstrated the robustness of this general strategy for targeted, programmable point mutation introduction.

### Adenine base editors

Using CBEs as a model, researchers sought to expand the base editor toolbox to include ABEs, which would use adenosine deamination chemistry to install A•T to G•C base pair conversions using an inosine-containing intermediate. ABEs would be capable of correcting the most common pathogenic single nucleotide variant (SNV), making them a vital tool for therapeutic genome editing [6,7]. While the general approach of replacing rAPOBEC1 for an ssDNA-specific adenosine deaminase enzyme was simple and elegant, unfortunately, no such naturally occurring enzyme existed, and it therefore needed to first be created.

As a first step, several RNA adenosine deaminase enzymes were installed into the CBE architecture in place of rAPOBEC1 and assessed for A•T to G•C activity levels. With no activity observed, researchers began the arduous process of using directed evolution to create an ssDNA-specific adenosine deaminase enzyme to produce the first ABE [8].

Directed evolution facilitates the enhancement or alteration of the activity of a given protein [9–11]. The protein of interest is mutagenized to produce a library of members, and active members are screened or selected to identify those with the new or enhanced activity of interest. To generate the first ABE, TadA, a tRNA adenosine deaminase sourced from *Escherichia coli*, which shares partial structural homology with the rAPOBEC1 enzyme employed by CBEs, was selected as a starting point. Over the course of seven rounds of directed evolution, ecTadA accumulated fourteen mutations to produce ABE7.10, which demonstrated on average 58% A•T to G•C editing efficiency across a variety of target sites with various sequence contexts [8]. It is important to note that adenine base editing did not require any BER inhibition components (such as the UGI of the CBE), presumably due to a lower efficiency of inosine excision by BER glycosylase enzymes. Consequently, no A•T to non-G•C editing was observed by ABE7.10.

### Limitations and modifications

We focus here on the limitations of base editing tools from a therapeutic perspective and the corresponding modifications to the original ABE and CBE constructs that have been engineered to overcome these limitations. The most obvious and major restriction of base editing technologies is the limited types of base pair conversions (C•G to T•A and A•T to G•C only) achievable with CBEs and ABEs. Expansion of the base editor toolbox in this area has been via the development of “glycosylase base editors,” which utilize the basic CBE architecture with additional enzyme components that facilitate excision of the uracil intermediate. Specifically, a suite of “CGBEs” (C•G to G•C base editors) has been developed, which exclude the UGI component of the CBE architecture and instead incorporate a uracil glycosylase enzyme and/or error-prone polymerases [12–16]. In these editors, the uracil intermediate is efficiently excised by either the endogenous uracil glycosylase enzyme of the cell, or that included in the CGBE architecture, to produce an abasic site. The resulting abasic site is then processed by the translesion synthesis pathway of the cell, or the polymerase included in the CGBE architecture, to mutagenize the target base, with a C•G to G•C base pair as the most common overall outcome. One such glycosylase base editor is currently being used in a clinical trial by Bioray Laboratories (discussed below). This same strategy was recently applied to ABEs as well, where an engineered hypoxanthine glycosylase enzyme (derived from N-methylpurine DNA glycosylase, MPG) was fused to an ABE, resulting in an adenine transversion base editor (AYBE) that mutagenizes target adenines, with an A•T to C•G base pair as the most common overall outcome [17].

An additional major limitation of early base editors was their targeting scope. Due to the restrictive editing window (positions 4 through 8 in the most widely used editors), many times a requisite PAM sequence could not be located at the necessary location. After establishing the architectural framework of the first CBE, subsequent efforts found that replacing the Cas9 enzyme with Cas9 variants with relaxed or altered PAM requirements, or Cas homologs from different species, resulted in editors with high editing efficiencies and significantly increased the targeting scope [18,19]. With the advent of extremely PAM-relaxed Cas9 variants, such as Cas9-NG and SpRY-Cas9, base editor targeting scope issues have been largely alleviated [20,21]. ABE7.10 was not as compatible with alternative Cas proteins, but this issue was resolved with the development of next-generation ABEs (discussed next).

An important characteristic of a therapeutic genome editor is high editing efficiency. Additional directed evolution efforts have been undertaken on both CBEs and ABEs to improve their overall efficiencies and remove sequence context biases that the deaminases possessed. Architectural engineering efforts on the original BE3 construct produced BE4, which has higher editing efficiencies and product purities than BE3 [22]. In fact, BE4 is currently being used in a clinical trial by Great Ormond Street Hospital for Children (GOSH; discussed below). Directed evolution efforts have also produced optimized CBEs via the improvement of deaminase kinetics and/or solubility [23,24]. Additionally, codon optimization is crucial for optimizing expression of BEs in different cell types, which is an important consideration therapeutically [25,26]. The further directed evolution of ABE7.10, resulting in various ABE8 and ABE9 constructs, was particularly important from a therapeutic context, as the resulting ABE8 variants are being used in the current clinical trials [25,27,28]. As mentioned previously, these ABE8 variants are also compatible with additional Cas homologs, which in effect expanded the targeting scope of these editors significantly. In fact, ABE8 variants are currently being used in two clinical trials (discussed below).

Finally, arguably the most important limitation of base editors from a therapeutic perspective are unintended edits. Unintended edits include any modification to the cell’s genome other than the intended edit. These may include “bystander edits” (which occur within the same protospacer as the intended edit), the wrong type of edit being installed at the target nucleotide (such as C•G to non-T•A conversions by CBEs) or “off-target edits” (which occur at other genomic loci in the cell), and it is important to note that these unintended editing events aren’t necessarily deleterious, and in fact many times can be benign. Bystander editing occurs as a consequence of deaminase processivity; if multiple target Cs or As are accessible within the ssDNA window, the deaminase will modify some or all. However, extensive deaminase engineering efforts have resulted in less-active deaminases that have narrower activity windows. Additionally, alteration of the overall architecture can manipulate the activity window. Furthermore, with the development of PAM-relaxed Cas9 variants, multiple gRNAs can be designed for a given target base, some of which will “push” the bystander bases outside of the editing window. These efforts are more thoroughly outlined in several key publications [22,25,29–31]. An additional type of bystander editing was observed with ABEs, namely, cytidine deamination activity by the mutant TadA protein, which would result in undesired bystander C•G to T•A mutations in addition to the desired A•T to G•C mutation [32]. This activity was then significantly reduced through engineering efforts, resulting in more precise ABE variants [33].

Extensive work has been done to characterize the off-target editing efficiencies of base editors, and three different types have been observed: gRNA-dependent off-targets; gRNA-independent DNA off-targets; and gRNA-independent RNA off-targets [34–38]. gRNA-dependent off-target editing occurs when Cas9 binds to a homologous genomic locus despite mismatches between the protospacer and spacer. The use of “high fidelity” Cas variants, which have lower tolerance for mismatches, can be incorporated into the base editor architecture to eliminate these [39]. Additionally, judicious choice of the gRNA can sometimes eliminate potential off-targets. gRNA-independent off-target editing occurs when the deaminase has access to ssRNA (both ABE and CBE) or ssDNA (CBEs only) within the cell (such as mRNA and transcription or replication bubbles) and deaminates cytosines or adenines within these bubbles. Several key publications have reported engineering of the deaminase domain (in both ABEs and CBEs) to reduce or eliminate RNA off-target editing events [40–43]. To reduce DNA off-target editing events, researchers have mutated the rAPOBEC1 protein to reduce its catalytic activity, as well as identified APOBEC homologs that naturally have lower gRNA-independent off-target editing activities [44,45]. Additionally, researchers have leveraged the previously undesired cytidine deamination activity observed with ABEs to engineer TadA-derived CBEs that have no gRNA-independent off-target DNA editing activity, like their ABE counterparts [46–48].

Notably, delivery of base editors as mRNA rather than plasmid DNA significantly reduces all forms of off-target editing [28]. A relationship between genome editing specificity and delivery modality/dosage was discovered prior to the development of base editors [49–52]. Genome editing agents typically modify the on-target locus first and will then modify off-target loci if their intracellular lifetime is long enough. To balance high on-target editing with minimal off-target editing, a short burst of a high level of active editor complex is therefore desired. Delivering DNA encoding for the editor will result in long-term expression, increasing chances of off-target editing. The lifetime of RNA is shorter than that of DNA, and transcription is not required to produce active editor when delivering mRNA encoding for the editor and gRNA. This results in a shorter timeframe between delivery and editing for mRNA and gRNA versus DNA, as well as shorter-term expression of active editor. Both mRNA and gRNA can be chemically modified to extend their half-lives as well. In a recent example of ex vivo base editing in hematopoietic stem cells (HSCs), chemically modified mRNA encoding BE3 and synthetic gRNA were electroporated, and BE3 protein expression peaked at 12-hour post-electroporation and was nearly entirely gone by 24-hour post-electroporation [53]. Furthermore, delivery of genome editing agents as purified protein:gRNA complexes (discussed below) results in the shortest overall lifetime of active editing agents. However, large-scale production of base editors at the purity required for therapeutic applications has been challenging, and thus mRNA delivery of base editors is generally preferred [54]. While off-target DNA edits are a therapeutic concern (particularly if they happen to occur in oncogenes or tumor suppressor genes), the quick turnover of mRNA within the cell alleviates some concerns regarding RNA off-targets.

## Base editor therapeutics

**Fig 2:**
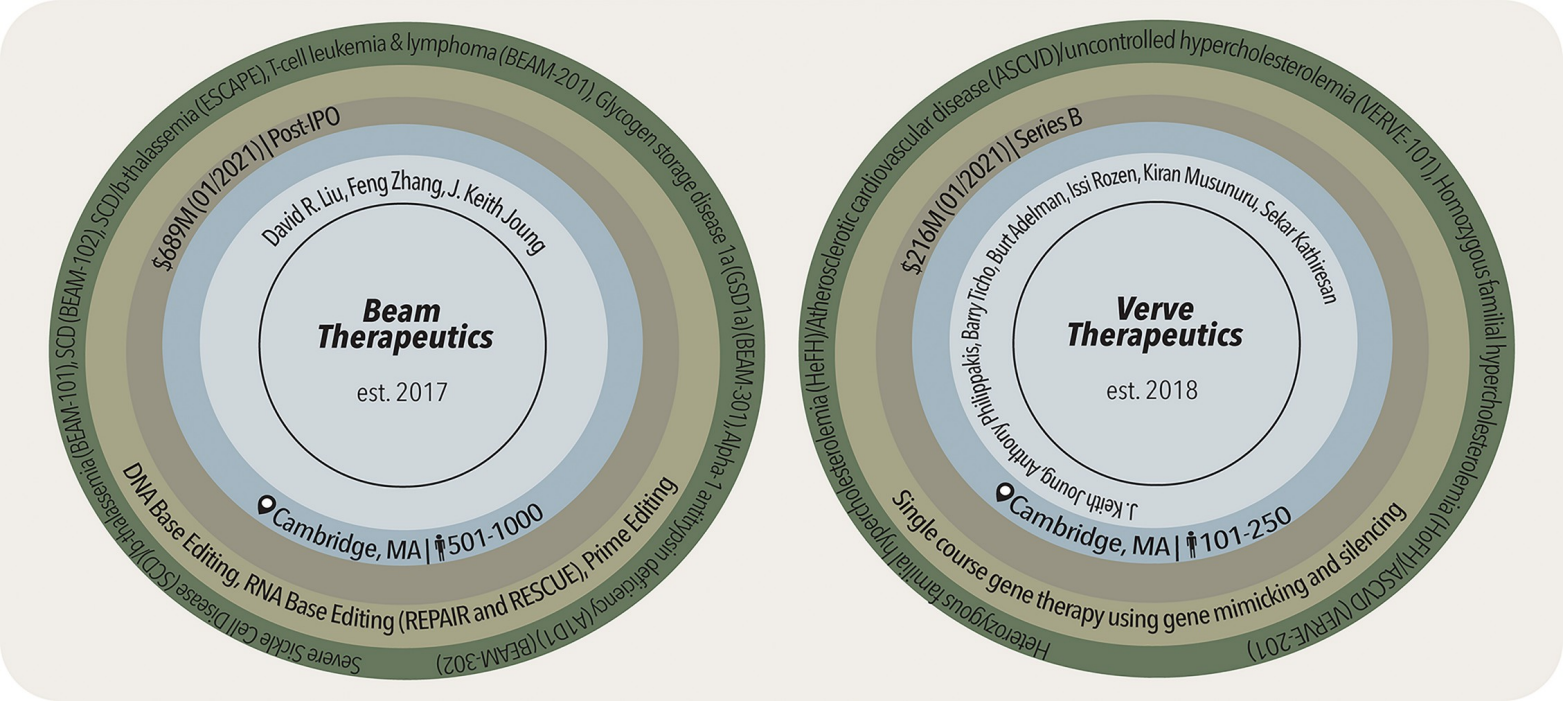
Profiling Beam Therapeutics and Verve Therapeutics. Starting with the inner most ring and moving outward, these profiles include the year each company was established, key scientific founders, location of headquarters and number of employees, last reported total funding and round acquired, technology specialization, and targeted indications addressed with the company’s specialized technology. Defined clinical trial candidates are denoted in parentheses. **Left**: profile on Beam Therapeutics. REPAIR is RNA editing for programmable A-to-I replacement and RESCUE is RNA editing for specific C-to-U exchange. **Right**: profile on Verve Therapeutics. Additional profiles can be found within S1 Table.

Translating the broad efforts of base editor development, mentioned above, into the clinical space requires an influx of support. To this end, many biotechnology companies have been founded or have sublicensed key base editor intellectual property since the development of the inaugural CBE to accomplish this lofty goal, with Beam Therapeutics and Verve Therapeutics dominating the base editor clinical trial space in the United States (Fig 2 and S1 Table). In the following discussions, we will detail the first cohort of base editor clinical trials, examining the targeted indications, delivery methods, and reported rates of success.

### Delivery options

Translating optimized base editing tools to the clinic requires viable delivery strategies, which has long been a bottleneck in the field of gene therapy. A variety of delivery strategies exist, with the choice of which one to use entirely dependent on the disease that is being treated. Delivery modalities can be roughly broken down by whether treatment will occur in vivo or ex vivo. In the case of in vivo delivery, the base editor is delivered directly into the target tissue(s) of the patient, while in the case of ex vivo delivery, cells are extracted from the patient, treated with the desired base editor, and subsequently redelivered into the patient via autologous transfer. Both strategies have a unique set of risks, challenges, and advantages. We expand on several relevant delivery avenues below.

In vivo gene editing must be used in cases where the treatment is designed to address a genetic disease afflicting an internal organ (i.e., the lung or liver). Given that genetic modification takes places within the body, in vivo therapies are subject to metabolic clearance and native immune responses [55]. Given these considerations, in vivo base editing treatments must be dosed such that editing efficiencies are high, yet toxicity and undesired immune responses are avoided/minimized. Given these requirements, viral vectors have historically been considered attractive delivery vehicles, despite their strict cargo packaging capacity limits. This has led to additional base editor modifications and optimizations to minimize their size. Specifically, split-intein base editors have been generated in which the base editor is split into two separate constructs (each packaged within its own virus), which are reassembled via intein chemistry when the separate halves are translated within the same cell [56–58]. Furthermore, base editors have been engineered using small Cas proteins to reduce the size of the full base editor construct [59–61]. These advances have leveraged adeno-associated viruses (AAVs) as the delivery vehicle, as AAVs posit the lowest immunological profile out of the suite of viral candidates for human in vivo delivery [62]. Unfortunately, one of the first reported in vivo clinical trials using an AAV resulted in a fatal immune response [63,64].

Circumventing potentially dangerous immuno-side effects can be achieved using nonviral delivery vehicles such as a lipid nanoparticles (LNPs), inorganic nanoparticles, or polymer-based nanoparticles [65]. In addition to an increased safety profile, these vehicles do not have restrictive size limitations and can be synthetically produced with relative ease compared to viral production [29,66,67]. These nanoparticles can be packaged with DNA or mRNA encoding the base editor and gRNA, or purified base editor:gRNA ribonucleoprotein (RNP) complex, which provides some flexibility. However, systemic treatment is difficult to achieve using nanoparticles, which is quite limiting. Typically, systemic delivery of LNPs (the most commonly used nanoparticle for in vivo nucleic acid delivery) results in preferential accumulation in the liver and spleen [68]. This biodistribution profile has been leveraged in Verve’s base editor clinical trial (discussed below). Nanoparticles can also be locally injected into certain organs, such as the inner ear [69,70].

Ex vivo genome editing is particularly well suited for treating blood disorders, such as hemoglobinopathies and leukemias. In addition to largely bypassing immune response issues, as genetic modification occurs outside of the patient, with ex vivo therapies, cells can be quality checked for accuracy before autologous transplantation [71]. While viral vectors can be used to deliver base editors ex vivo, nucleic acid or RNP electroporation is also an option. This method is quite efficient and, similar to in vivo nanoparticles, does not have payload size restrictions. Unsurprisingly, these advantages are leveraged in three of the four clinical studies discussed below.

### Framework for a clinical trial

**Fig 3:**
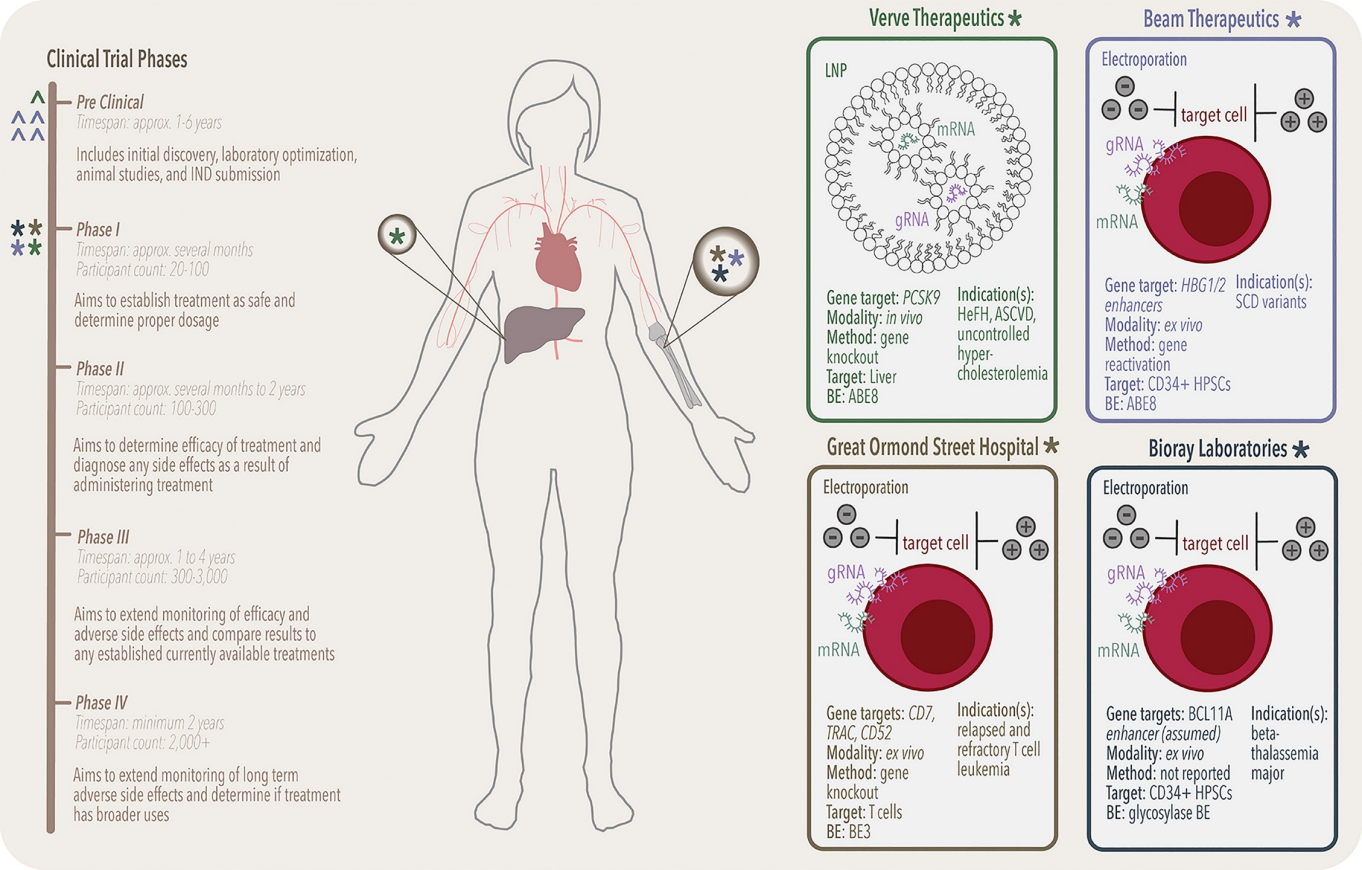
Overview of current base editing clinical trials. **Left**: a simplified timeline for the path of a drug from preclinical to FDA approval. Current statuses of ongoing trials are represented by colored special characters: ^ are clinical candidates in development, while * are candidates currently undergoing clinical trials sponsored by Verve Therapeutics (forest green), Beam Therapeutics (mauve), Great Ormond Street Hospital (mustard), and Bioray Laboratories (navy). **Middle**: a visual representation of the target of each of the four clinical trials; a single in vivo treatment is delivered to the liver (forest green) and three others function by ex vivo treatments, which are then subsequently readministered via bloodstream IV and will repopulate cells in the bone marrow (mauve, mustard, navy). **Right**: detailed breakdown of the four ongoing clinical trials. Shown are the delivery modality (mRNA (teal)/gRNA (purple) electroporation versus lipid nanoparticle delivery), as well as targeted indications.

Once a proposed therapeutic has been put through rigorous testing and optimization, the transition from the preclinical to clinical phase (I to IV) begins (Fig 3). It is important to recognize the strict demands these companies face in bringing a drug candidate to the clinical trial phase. The United States Federal Food and Drug Administration (USFDA) oversees all clinical trials in the US to ensure the safety and welfare of trial participants. Strict regulations are put in place for each phase of a clinical trial to maintain the integrity of the study. These regulations are for all levels of trial involvement: design, management and handling, data analysis, data reporting, and overall good practice.

## Ongoing base editor trials

### VERVE-101

In July 2022, Verve Therapeutics announced the first patient had been dosed with VERVE-101, an investigational in vivo base editing medicine targeting *PCSK9* (Fig 3). The clinical trial, which is taking place in New Zealand and the United Kingdom (NCT05398029), marks the first instance of a base editor treatment in human patients. Another noteworthy aspect of the clinical trial is that the base editing occurs in vivo (rather than ex vivo), which is a significant milestone. VERVE-101 is an intended treatment for heterozygous familial hypercholesterolemia (HeFH), atherosclerotic cardiovascular disease (ASCVD), and uncontrolled hypercholesterolemia. In HeFH, the liver’s ability to metabolize low-density lipoprotein (LDL) is compromised [72]. The buildup of LDL within the body results in high cholesterol levels that in turn form plaques, which, over time, will cause arteries to harden and restrict blood flow. This often leads to coronary artery disease and potentially fatal myocardial infarctions. *PCSK9* is a target to lower LDL levels and treat HeFH, as the PCSK9 protein degrades the LDL receptor, which is required for uptake of LDL particles by hepatocytes. Additionally, naturally occurring loss-of-function mutations in *PCSK9* have been identified in healthy individuals [73,74].

VERVE-101 is a single-course treatment for HeFH that will permanently knock out *PCSK9* in the liver to reduce LDL levels. This is achieved using an ABE8 variant to mutate the GT (the target A is base paired with the underlined T) splice donor at the exon 1/intron 1 boundary in *PCSK9* [75]. Following A•T to G•C point mutation introduction, intron 1 is retained in the mature mRNA transcript, resulting in a premature stop codon and degradation of the mRNA through nonsense-mediated decay [28,76,77]. VERVE-101 is administered via an intravenous infusion of an engineered LNP containing ABE8-encoding mRNA and the *PCSK9*-targeting gRNA, resulting in LNP delivery mainly to the liver of patients [75,78,79]. This approach was recently undertaken in cynomolgus monkeys, where 90% reduction of PCSK9 levels in the blood was observed [75]. In this Phase Ib trial, Verve seeks to assess the safety and pharmacodynamic profile of VERVE-101.

### BEAM-101

In July 2022, Beam Therapeutics announced patient enrollment had begun for its BEACON trial (NCT05456880), which aims to assess its BEAM-101 therapy as a treatment for three forms (HbSS, HbSβ^0^, and HbSβ^+^) of severe sickle cell disease (SCD) (Fig 3). These three types of SCD are all caused by mutations in the hemoglobin β subunit (*HBB*) gene, which encodes for the β-globin protein [80]. The most common form of hemoglobin in adults, hemoglobin A (HbA), is a tetramer comprised of two β-globin subunits and two α-globin subunits. All three forms of SCD that BEAM-101 is intended to treat have the “HbS” mutation in one of the *HBB* alleles, which is an A•T to T•A mutation that causes a Glu6Val substitution in the β-globin protein. This hydrophobic amino acid substitution causes β-globin proteins to “stick” to each other and polymerize to form long fibers. These polymers in turn distort the shape of erythrocytes, causing “sickling” of the cells. Individuals with the HbSS form of SCD are homozygous for this mutation (this is known as “sickle cell anemia”). Individuals with the HbSβ^0^ and HbSβ^+^ forms of SCD have the HbS mutation on one allele and another mutation in *HBB* on the other allele that impacts expression of the β-globin protein. Those with HbSβ^0^ have no expression of β-globin from this second allele, and those with HbSβ^+^ have reduced production of β-globin from this second allele [81]. In all three forms of SCD, the red blood cells become sickled, which causes blood flow clogs [82]. This consequently results in sickle cell crises (attacks of pain), infections, and stroke.

Beam’s approach to treat SCD is to “reactivate” expression of fetal hemoglobin (HbF), which is comprised of two α-globin subunits and two γ-globin subunits. HbF is involved in transporting oxygen in fetuses, and expression of γ-globin (encoded by *HBG1* and *HBG2*, which encode the same protein but have different regulatory sequences) naturally decreases to very low levels within a year of birth. Reactivation of HbF can compensate for low levels of β-globin and inhibit polymerization of HbS proteins [83]. Certain healthy individuals naturally have mutations that cause hereditary persistence of fetal hemoglobin (HPFH), in which HbF levels in adults exceed the normal level. BEAM-101 is an autologous cell therapy that seeks to introduce the “British” HPFH mutation (a T•A to C•G mutation in the *HBG1* and *HBG2* enhancers) into patient-derived hematopoietic stem and progenitor cells (HSPCs) ex vivo [84,85]. Specifically, CD34^+^ HSPCs are harvested from the patient and electroporated with ABE8-encoding mRNA and *HBG1/2*-targeting gRNA. The resulting mutation prevents the BCL11A repressor from binding to the *HBG1/2* enhancers. To facilitate efficient engraftment of the edited cells, patients must be conditioned prior to reintroduction of the edited cells. Beam has previously reported the successful, high-efficiency editing and subsequent robust reactivation of HbF in ex vivo-edited patient-derived CD34^+^ HSPCs [28]. In this Phase I/II trial, Beam seeks to assess the safety and efficacy of BEAM-101.

### BE-CAR7

In May 2022, GOSH, in collaboration with University College London (UCL), began patient enrollment for its BE-CAR7 trial (NCT05397184), which aims to assess the safety of this treatment for relapsed and refractory T cell leukemia in patients aged 6 months to 16 years (Fig 3). T cells (a type of white blood cell) are derived from HSCs in the bone marrow and differentiate into T cells in the thymus. Certain genetic and epigenetic modifications can occur during this process and cause T cell acute lymphoblastic leukemia (T-ALL), which is an aggressive and quick-progressing leukemia [86,87]. Chimeric antigen receptor (CAR)-T cell therapy has emerged as a promising treatment for such types of cancer. CAR-T cell therapy involves collecting T cells from either a healthy donor (allogeneic CAR-T cell therapy, which the BE-CAR7 trial is) or the patient (autologous CAR-T cell therapy) and engineering the cells to express a CAR on the cell surface (this is generally achieved using lentiviral transduction methods). The CARs are receptor proteins that both bind to a specific antigen that the leukemia cells are expressing and activate T cell function. In the case of BE-CAR7, the T cells are engineered to express a CAR that recognizes CD7, a transmembrane protein that is highly expressed on both normal and malignant T cells [88]. The resulting CAR7 cells can in theory then be infused into the patient, where they will bind to CD7-expressing malignant T cells and destroy them.

Unfortunately, both the engineered CAR-T cells and the malignant T cells express CD7, resulting in CAR-T cell “fratricide,” in which the CAR-T cells target and destroy themselves. To prevent this, the endogenous CD7 gene must first be knocked out. Additionally, the T cell receptor α chain (TRAC) gene must also be knocked out to prevent graft-versus-host disease (which occurs when donor T cells recognize the patient’s cells as foreign and destroy host tissue). Finally, the CD52 gene must also be knocked out, to enhance the lifetime of the CAR-T cells in the presence of the lymphocytic leukemia medication alemtuzumab (which is an antibody that binds to CD52). Therefore, in the BE-CAR7 trial, prior to lentiviral transduction of the CAR7, the T cells are electroporated with CBE-encoding mRNA (specifically, BE3) and three synthetic gRNAs, which target *CD7*, *TRAC*, and *CD52* for knock-out. The *CD7*-targeting gRNA targets the CBE to a Gln codon (CAG codon) in *CD7* and converts it to a premature stop codon (TAG) via C•G to T•A base editing, resulting in nonsense-mediated decay of the mRNA transcript and knock-out of the gene. It should be noted that bystander mutations are also concurrently introduced but are benign due to knock-out of the gene. *TRAC* and *CD52* knock-out is accomplished similarly. Multiplexing gene knock-outs using traditional, DSB-reliant genome editing methods is accompanied by large-scale chromosomal rearrangements and cytotoxicity, which are avoided when using base editors to install premature stop codons or splice site disruptions [89]. Therefore, future CAR-T cell therapies requiring multiplexed knock-out strategies will greatly benefit from the use of base editors.

The GOSH and UCL team recently reported specific cytotoxicity of engineered *CD7* knock-out CAR-T cells against CD7^+^ T-ALL cells both in vitro and an in vivo humanized mouse model [53]. In this Phase I trial, the team seeks to assess the safety of the BE-CAR7 treatment and assess if the CAR7 T cells can eliminate T cell leukemia. In exciting recent news, Alyssa, the first patient to be administered BE-CAR7, reported complete remission of T-ALL six months after her treatment.

### BRL-103

In July 2022, Bioray Laboratories announced its BRL-103 clinical trial (NCT05442346), which is an autologous cell therapy for patients with β-thalassemia major (Fig 3). β-Thalassemias, similar to SCD, are caused by mutations in *HBB* that cause reduced or no expression of β-globin. β-Thalassemia major is caused by mutations in both *HBB* alleles and symptoms typically include severe anemia. Without treatment, patient death typically occurs before age 20. Treatment includes periodic blood transfusions and chelation of iron overload that is caused by the repeated blood transfusions.

BRL-103 is similar to BEAM-101 and involves harvesting HSCs from patients, reactivating HbF using base editing, and reintroducing the edited cells into the patients after conditioning. A major distinction from the BEAM-101 trial is BRL-103’s use of a glycosylase base editor, which presumably is used to mutate the *BCL11A* enhancer (based on similarities to their NCT04211480 clinical trial) [90]. As mentioned previously, the BCL11A repressor silences *HBG1/2* expression. Disruption of *BCL11A* expression would therefore reactivate *HBG1/2* expression. The use of a base editor to mutate the *BCL11A* enhancer rather than wtCas9 has a variety of benefits including fewer genotype outcomes, lower risk of chromosomal rearrangements due to DSBs, and lower cytotoxicity. While no publications have been reported on BRL-103 yet, preliminary results from their Phase I/II clinical trial NCT04211480 in which Cas9 was used to mutate the *BCL11A* enhancer have been published and showed increased hemoglobin production and a high persistence of edited cells in the bone marrow [90]. In this Phase I/II trial, Bioray seeks to assess the safety and efficacy of BRL-103.

### Clinical expansions of current trials

In addition to the VERVE-101 and BEAM-101 clinical trials, other base editor-based therapies are earlier in the clinical pipeline from both companies. For Verve, their second drug, VERVE-201, targets *ANGPTL3* in the liver for permanent silencing. This treatment is for individuals with homozygous familial hypercholesterolemia (HoFH). In theory, this treatment could also be used for patients with HeFH who do not receive sufficient results from the *PCSK9* therapy. VERVE-201 is still preclinical in the investigational new drug (IND) enabling phase but is expected to be rolled out in the clinic in 2024. The news of this development accompanied reporting that the VERVE-101 clinical trial in the US has been put on hold; however, studies are still ongoing in New Zealand and the UK.

Similarly, Beam has forged ahead on several new drugs: ESCAPE-1 in which ex vivo multiplexed editing of *HBG1/2* and *CD117* can treat SCD and β-thalassemia with less toxic conditioning of the patient; BEAM-201 in which multiplexed gene knock-out will be used for T-cell leukemia and lymphoma treatment; BEAM-301 in which in vivo correction of the R83C mutation in *G6PC1* in the liver will be used to treat glycogen storage disease 1a; and BEAM-302 in which in vivo gene correction of the E342K mutation in *SERPINA1* will be used to treat alpha-1 antitrypsin deficiency. ESCAPE-1, BEAM-201, and BEAM-301 are all in the IND enabling phase, while BEAM-302 is still relatively early in the optimization phase. It is important to note that additional therapies are also in development at both Verve and Beam; however, for proprietary reasons, further details on these technologies are not presently available.

## Ethical implications and future of the field

Given the fast pace of genome editing therapeutics, and the quick turnaround time from the development of the first base editor to base editor clinical trials, ethical discussions and considerations are imperative. This is particularly timely given the events of 2018, when CRISPR/Cas9 was used to perform germline genome editing on two embryos, causing members of the general public to feel mistrust and apprehension about therapeutic genome editing in general. Therefore, transparency and open discussions among scientists, bioethicists, policy makers, clinicians, and patient advocacy groups is necessary to ensure productive progress forward and avoid the dissemination of misinformation. Given the short timespan from base editor discovery in 2016 to the initiation of clinical trials now, it is also important to expand our basic understanding of how base editors function, which will ultimately aid base editor drug development and potentially clinical approval.

In addition to the candidate therapies in clinical trials underway, more work is being done to expand the host of potentially curative genomic medicines. These efforts are both inside and outside the base editor field. For example, prime editors are one such next step in the evolution of genomic medicine and have addressed some of the limitations of base editors [91]. This new technology, like base editors, avoids the use of DSBs and therefore installs genomic modifications with high precision. Prime editors perform genome editing using a completely different mechanism than base editors, and the two technologies are therefore complementary to each other. Prime editors employ a reverse transcriptase (RT) fused to nCas9 and an extended gRNA, called a prime editing gRNA (pegRNA) that has a 3′ extension. The pegRNA encodes both the location of editing (via the spacer sequence) and the edit to be introduced (via the 3′ extension). Following DNA binding and nicking of the PAM-containing strand, the RT directly appends a portion of the 3′ extension of the pegRNA sequence onto the broken DNA end. In this manner, prime editors can install any type of small modification into the genome in a programmable and precise manner. The quick establishment of Prime Medicine to develop prime editors into therapeutics is a sign of additional exciting clinical trials in the future. Despite the uncertainty and ambiguity of scientific research, one thing is certain: Base editors are a staple of genomic medicine and will clearly have a real impact on society and human health.

Exciting new work within the base editor field has yielded mitochondrial genome editing agents, which have the potential to cure genetic disorders caused by mitochondrial mutations [92]. Mitochondrial genome editing had been unfeasible until recently, for reasons related to mitochondrial DSB repair and delivery of nucleic acids to the mitochondria. Reliable nucleic acid delivery to the mitochondria has not yet been established [93]; thus, CRISPR-based genome editing agents (which require gRNAs) cannot be used for mitochondrial genome editing. CRISPR-free programmable nucleases such as transcription activator-like effector nucleases (TALENs) and zinc finger nucleases (ZFNs) can be delivered to the mitochondria via the use of mitochondrial targeting signals (MTSs). However, cleaved mitochondrial DNA is degraded rather than repaired; thus, precision mitochondrial genome editing cannot be performed by TALENs and ZFNs [94–96]. Therefore, TALE- and ZF-derived CBE and ABEs were developed to enable mitochondrial genome editing [97–101]. As these editors are fully protein based, they can be delivered to the mitochondria, and as they use uracil and inosine intermediates, they install point mutations into mitochondrial DNA rather than degrade it. This new class of base editors opens up new therapeutic opportunities in the mitochondrial disorder space.

## Conclusions

The fast timeline (6 years) for the progression of base editors from bench to bedside was supported by the concurrence of several factors, including the robustness of the technology, an influx of support to both academic research on base editors, as well as to the biotechnology sector, and the knowledge gained from therapeutic efforts on other genome editing agents, particularly in the area of delivery. Fervent research in the space of base editing uncovered limitations of the technology (including undesired editing events) almost as quickly as it developed solutions to these limitations, allowing for the evolution of base editors from research tools to therapeutic agents. We described here this development, and the four current examples of base editing clinical trials. With several more on the horizon, we are excited to see additional creative applications of these technologies to human health.

## Supporting information

S1 Table Sampling of prominent biotechnology companies within the gene editing therapeutics field. Information presented is an extension of Fig 2, across a broader range of biotechnology companies. In the interest of space, the following set of exclusionary criteria was used to determine the final list of companies represented: non-CRISPR or base editor-based technology, not based in the US, acquired by a larger company (larger, acquiring companies may be included), solely cell therapy–focused, large-scale pharmaceutical companies, and nonhuman based research applications.
(PDF)

## Abbreviations

AAV: adeno-associated virus
ABE: adenine base editor
APOBEC: Apolipoprotein B mRNA editing enzyme, catalytic polypeptide
ASCVD: atherosclerotic cardiovascular disease
BER: base excision repair
CAR: chimeric antigen receptor
CBE: cytosine base editor
CRISPR: clustered regularly interspaced short palindromic repeats
dCas9: “dead” Cas9
DSB: double-strand break
GOSH: Great Ormond Street Hospital for Children
gRNA: guide RNA
HbA: hemoglobin A
HbF: fetal hemoglobin
HeFH: heterozygous familial hypercholesterolemia
HoFH: homozygous familial hypercholesterolemia
HPFH: hereditary persistence of fetal hemoglobin
HSC: hematopoietic stem cell
HSPC: hematopoietic stem and progenitor cell
IND: investigational new drug
LDL: low-density lipoprotein
LNP: lipid nanoparticle
MTS: mitochondrial targeting signal
nCas9: nickase Cas9
PAM: protospacer adjacent motif
pegRNA: prime editing gRNA
RNP: ribonucleoprotein
RT: reverse transcriptase
SCD: sickle cell disease
SNV: single nucleotide variant
spCas9: *Streptococcus pyogenes* Cas9
TALEN: transcription activator-like effector nuclease
T-ALL: T cell acute lymphoblastic leukemia
TRAC: T cell receptor α chain
UCL: University College London
UGI: uracil glycosylase inhibitor
USFDA: United States Federal Food and Drug Administration
ZFN: zinc finger nuclease
