# Supplementary material for: In the business of base editors: Evolution from bench to bedside
Source: PLoS Biol. 2023 Apr 12;21(4):e3002071. doi: 10.1371/journal.pbio.3002071 (PMC10096463; doi:10.1371/journal.pbio.3002071)
Supplement: S1 Table — Information presented is an extension of Fig 2, across a broader range of biotechnology companies. In the interest of space, the following set of exclusionary criteria was used to determine the final list of companies represented: non-CRISPR or base editor-based technology, not based in the US, acquired by a larger company (larger, acquiring companies may be included), solely cell therapy–focused, large-scale pharmaceutical companies, and nonhuman based research applications. (PDF) [file pbio.3002071.s001.pdf]

Supporting Information for

In the Business of Base Editors: Evolution from Bench to Bedside

Elizabeth M. Porto and Alexis C. Komor

S1 Table	Sampling of prominent biotechnology companies within the gene editing therapeutics field

S1 Table: Sampling of prominent biotechnology companies within the gene editing therapeutics field. Information presented is an extension of **Figure 2**, across a broader range of biotechnology companies. In the interest of space, the following set of exclusionary criteria was used to determine the final list of companies represented: non-CRISPR or BE based technology, not based in the United States, acquired by a larger company (larger, acquiring companies may be included), solely cell-therapy focused, large-scale pharmaceutical companies, and non-human based research applications.

Company	Year Founded	Founders	Headquarters	No. of Employees	Amount and Source of Funding	Technology Specialization	Targeted Indications
Acrigen Biosciences	2019	Joe Bondy-Denomy & David Rabuka	San Francisco, CA	1-10	$1.2M Grant round (02/2022)	AcrTAIN software platform to identify new Cas enzymes and combine with engineered recombinant anti-CRIPSR (ErAcr) proteins to minimize off-target editing	No declared focus
Arbor Biotechnologies	2016	Feng Zhang, David R. Walt, David Scott, Winston Yan	Cambridge, MA	51-100	$230M Series B (11/2021)	Engineered nucleases (DNA, RNA, and transposases)	Primary hyperoxaluria, liver diseases 1&2
Artisan Bio	2019	Tanya Warnecke & Ryan Gill	Denver, CO	1-10	$21M Series A (11/2020)	STAR-CRISPR platform comprised of an enhanced nuclease and STAR gRNAs	Non-small cell lung cancer, pediatric sarcomas, multiple myeloma
Capsida Biotherapeutics	2019	Nicholas Flytzanis, Nick Goeden, Viviana Gradinaru	Los Angeles, CA	11-50	$140M Corporate round (04/2021)	Tissue targeted gene therapies using optimally engineered AAV capsids	Central Nervous System (CNS) disorders
Caribou Biosciences	2011	Jennifer Doudna, James Berger, Martin Jinkek, Rachel E. Haurwitz	Berkeley, CA	101-250	$168M Series C (03/2021)	CRISPR hybrid RNA-DNA genome editing (Cas12a chRDNA) and CAR-T/CAR-NK cell therapies	Hematologic, solid tumor, non-Hodgkin lymphoma, 2 undisclosed CAR-T programs
CRISPR Therapeutics	2013	Emmanuelle Charpentier, Chad Cowan, Daniel Anderson, Matthew Porteus, Rodger Novak, Shaun Foy	Cambridge, MA	101-250	$130M Series B (12/2020)	Traditional CRISPR/Cas9 approach for geneteically-defined diseases and cellular engineering	Beta-thalassemia, Sickle Cell Disease (SCD)
Editas Medicine	2013	Feng Zhang, George Church, J. Keith Joung, Jennifer Doudna	Cambridge, MA	101-250	$657M Post-IPO (01/2021)	Traditional Cas9 and Cas12a (Cpf1) for *in vivo* gene edited and *ex vivo* gene edited cellular medicines	Solid tumors, SCD, Beta-thalassemia, Leber congenital amaurosis 10 (LCA10)
ElevateBio (a LifeEdit company)	2017	David Hallal, Mitchell Finer, Vikas Sinha	Cambridge, MA	101-250	$845M Series C (03/2021)	Proprietary novel RNA-guided nucleases (RGNs) and BEs derived from AgBiome's colelction of microbes with broad range of PAMs	Neurological conditions with high unmet need
Emendo Biotherapeutics	2015	Shilo Ben Zeev	New York City, NY	11-50	$61M Series B (01/2020)	Novel OMNI nucleases with non-NGG PAMs to maximize activity and allele-specificity while minimizing off-target activity	Severe Congenital Neutropenia (SCN)
Excision BioTherapeutics	2015	Rob Simmons & Thomas Malcolm	Philadelphia, PA	11-50	$78M Grant round (09/2022)	Dual gRNA system to force large DNA deletions and precent viral escape and reproduction	Human immunodeficiency virus (HIV), Progressive multifocal leukoencephalopathy (PML), Herpes simplex virus (HSV), Hepatitis B virus (HBV)

Company	Year Founded	Founders	Headquarters	No. of Employees	Amount and Source of Funding	Technology Specialization	Targeted Indications
Graphite Bio	2020	Daniel Dever, Maria-Grazia Roncarolo, Matthew Porteus	San Francisco, CA	101-250	$196M Series B (03/2021)	UltraHDR platform aimed at 'find and replace' gene editing with HiFi Cas9	SCD and Beta-thalssemia
Hunterian Medicine	2016	Vinod Jaskula-Ranga	Cambridge, MA	1-10	$3.8M Grant round (05/2021)	Bidirectional promoter aimed at shrinking Cas protein for packaging within a single AAV capsid	No declared focus
iECURE	2012	Jim Wilson	Philadelphia, PA	11-50	$50M Series A (09/2021)	Proprietary ARCUS nuclease used for duplicate healthy gene insertion	Genetic liver diseases with emphasis on early childhood patients
Inscripta	2015	Andrew Garst, Ryan T. Gill, Tanya Warnecke	Denver, CO	101-250	$460M Series E (04/2021)	Further development on MAD7 nuclease for optimized viral delivery	No declared focus
Intellia Therapeutics	2014	Jennifer Doudna, Andy May, Derrick Rossi, Erik Sontheimer, Luciano Marraffini, Nessan Bermingham, Rodolphe Barrangou	Cambridge, MA	251-500	$925M Post-IPO (06/2021)	Traditional CRISPR/Cas9 genome editing for *in vivo* and *ex vivo* therapies	Transthyretin amyloidosis, hereditary angiodema, acute myeloid leukemia, Alpha-1 antitryp-sin deficiency (A1D1), Hodgkin's lymphoma
Ligandal	2014	Andre Watson & Christian Foster	Brooklyn, NY	1-10	$4.6M Seed round (02/2019)	Platform for creating target cell population-specific gene therapy delivery vehicles	COVID-19
Locanabio	2016	David Nelles & Gene Yeo	San Diego, CA	11-50	$156M Series B (12/2020)	CORRECTX platform aimed to create modular RNA binding protein systems for multi-functional RNAs	Neuro-degenerative, neuromuscular, and retinal diseases
Locus Biosciences	2015	Charles Gersbach, Chase Beisel, David Ousterout, Nick Taylor, Paul Garofolo, Rodolphe Barrangou	Morrisville, NC	11-50	$129M Series B (05/2022)	Technology mergining bacteriophage targeting with CRISPR-Cas3 activity to combat bacterial pathogens	Antibiotic-resistant infections, microbiome-related diseases, inflammatory bowel disease (IBD)
Mammoth Biosciences	2017	Jennifer Doudna, Lucas Harrington, Ashley Tehranchi, Janice Chen, Trevor Martin	San Francisco, CA	101-250	$265M Series D (09/2021)	Specializes in miniature Cas enzymes for ease of delivery and precise edits with alternative PAMs	No declared focus
Metagenomi	2018	Jillian Banfield & Brian Thomas	San Francisco, CA	101-250	$357M Corporate round (11/2022)	Developing next-generation gene editing tools using optimized, novel nucleases	Liver diseases, Hemophilia A, oncological diseases
Poseida Therapeutics	2014	Eric Ostertag	San Diego, CA	251-500	$411M Post-IPO (08/2022)	Proprietary Cas-CLOVER homo-dimer nuclease system for gene editing in resting T-cells	Hemophilia A, Ornithine transcarbamylase deficiency (OTC)

Company	Year Founded	Founders	Headquarters	No. of Employees	Amount and Source of Funding	Technology Specialization	Targeted Indications
Prime Medicine	2019	David Liu, Andrew Anzalone, Jeremy Duffield	Cambridge, MA	101-250	$315M Series B (07/2021)	Leverages prime editing technology for 'search and replace' gene editing	No declared focus
Scribe Therapeutics	2017	Jennifer Doudna, Benjamin Oakes, Brett Staahl, David F. Savage	San Francisco, CA	11-50	$120M Series B (03/2021)	X-editing technology built on novel CRISPR foundation for *in vivo* genetic modification	No declared focus
Sherlock Biosciences	2019	Feng Zhang, David R. Walt, Deborah Hung, Jim Collins, Jonathan Gootenberg, Omar Abudayyeh, Pardis Sabeti, Rahul Dhanda, Todd Golub	Cambridge, MA	11-50	$136M Series B (03/2022)	CRISPR-utilizing specific high-sensitivity enzymatic reporter unlocking (SHERLOCK) for single molecule detection of nucleic acid targets	No declared focus
Spotlight Therapeutics	2017	Alexander Marson, Jacob Corn, Patrick Hsu	San Francisco, CA	11-50	$81M Series B (03/2022)	Use of targeted active gene editors (TAGE) CRISPR effectors for direct delivery of cell-selective gene therapies	Opthalmic and HSC-mediated diseases and hemoglobino-pathies
Tessera Therapeutics	2018	Geoffrey von Maltzahn & Jacob Rubens	Cambridge, MA	101-250	$532M Series C (04/2022)	DNA- and RNA-based GENE WRITING technology	No declared focus
Tome Biosciences	2021	Omar Abudayyeh & Jonathan Gootenberg	Watertown, MA	51-100	$96M Series A (01/2022)	Licensed programmable addition via site-specific targeting elements (PASTE) technology, with future technology in development	No declared focus
Wave Life Sciences	2012	Takeshi Wada & Greg Verdine	Boston, MA	101-250	$174M Post-IPO (02/2018)	Stereopure oligonucleotides for range of modalities such as RNA editing	Huntington's disease, Duchenne muscular dystrophy (Dmd), A1D1
